# Supplementary material for: Environment Effects on X-Ray Absorption Spectra With Quantum Embedded Real-Time Time-Dependent Density Functional Theory Approaches
Source: Front Chem. 2022 Feb 28;10:823246. doi: 10.3389/fchem.2022.823246 (PMC8919347; doi:10.3389/fchem.2022.823246)
Supplement: Supplementary file 1 [file DataSheet1.PDF]

Supplementary Material for: Environment effects on XAS spectra with quantum embedded rt-TDDFT Environment effects on X-ray absorption spectra with quantum embedded real-time Time-dependent density functional theory approaches

1 SUPPLEMENTARY DATA, TABLES AND FIGURES

1.1 MO-bases analysis of excitations

Table S1. We report for the MO-based decomposition of the transition at $\omega = 668.634$ eV from a supermolecular calculation of $[F(H_2O)_8]^-$ at B3LYP/aug-cc-pvtz level of theory. The elements of the CI-vector, X_{ia}, corresponding to the occupied-virtual MO pair are also listed.

Excitation energy (eV)		
$\omega = 668.634$		
occ.	virt.	X_{ia}
1	46	-0.99431
1	47	-0.09308

```
Vector   46  Occ=0.000000D+00  E= 2.882190D-01
   Bfn.  Coefficient  Atom+Function         Bfn.  Coefficient  Atom+Function
  ----- ------------  ---------------      ----- ------------  ---------------
     5      1.110236   1 F  s                4     -0.642763   1 F  s
    17     -0.354755   1 F  pz              16      0.291654   1 F  py
    63      0.253890   8 O  px              76     -0.246397  14 O  s
    78      0.238905  14 O  py              71     -0.234837  11 O  py
    77     -0.202945  14 O  px              58      0.201848   5 O  pz
```

```
 Vector   47  Occ=0.000000D+00  E= 3.017870D-01
   Bfn.  Coefficient  Atom+Function         Bfn.  Coefficient  Atom+Function
  ----- ------------  ---------------      ----- ------------  ---------------
    16      0.694780   1 F  py              17     -0.688708   1 F  pz
     5     -0.480913   1 F  s               15      0.381587   1 F  px
    85      0.345763  17 O  py              83      0.296018  17 O  s
     4      0.277528   1 F  s               13     -0.240524   1 F  py
    48      0.240055   2 O  s               14      0.233456   1 F  pz
```

1.2 DSF and Fourier Transform of TD induced density

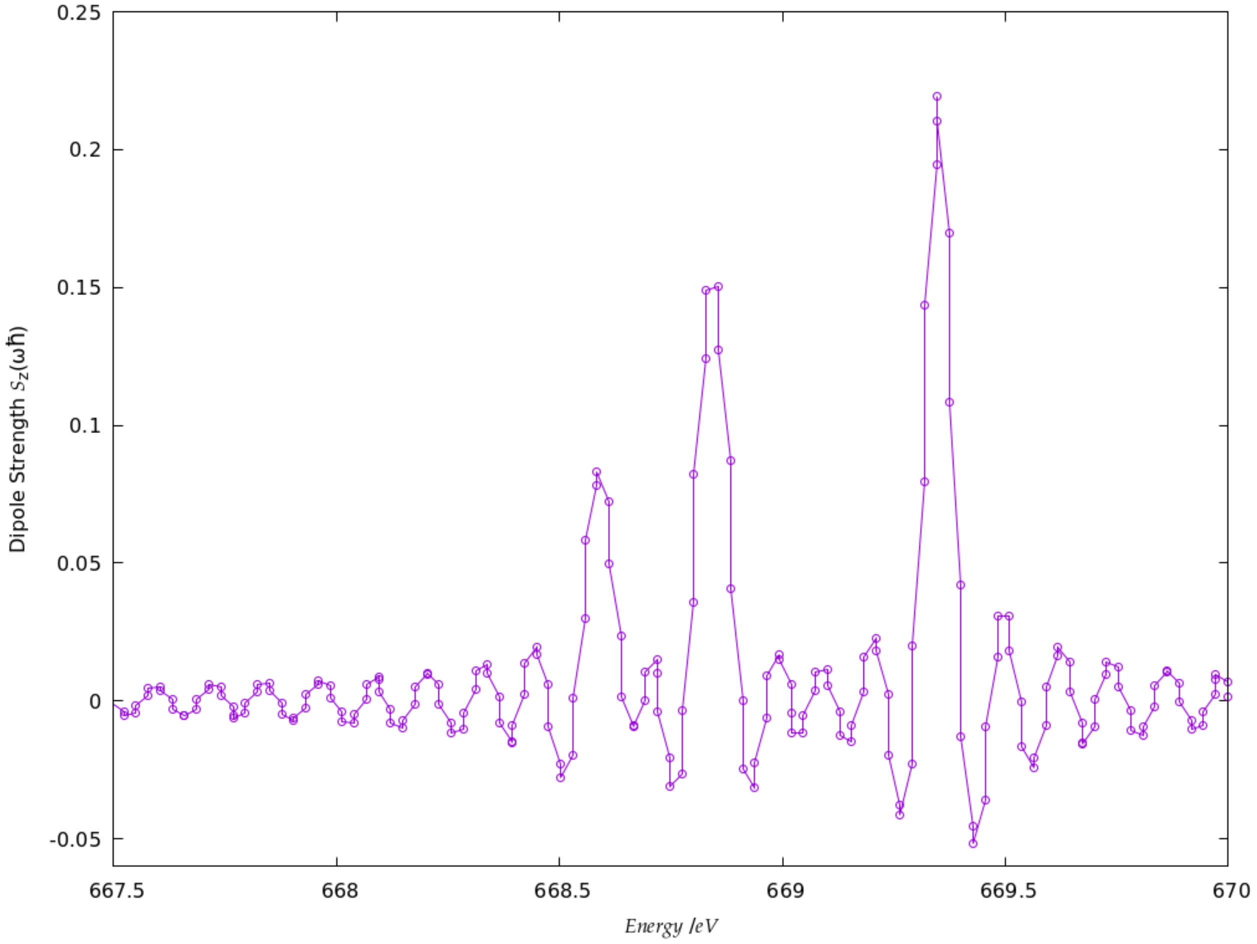

Figure S1. We report the z-direction contribution to the dipole strength function in th range 667-670 eV for $[F(H_2O)_8]^-$. The time-domain is represented by 56001 sampling points and prior to Fourier transformation was zero-padded in order to extend it up to 2×10^{19} sampling points. Despite the increased spectral density the DSF features a very low quality in the energy range of interest.

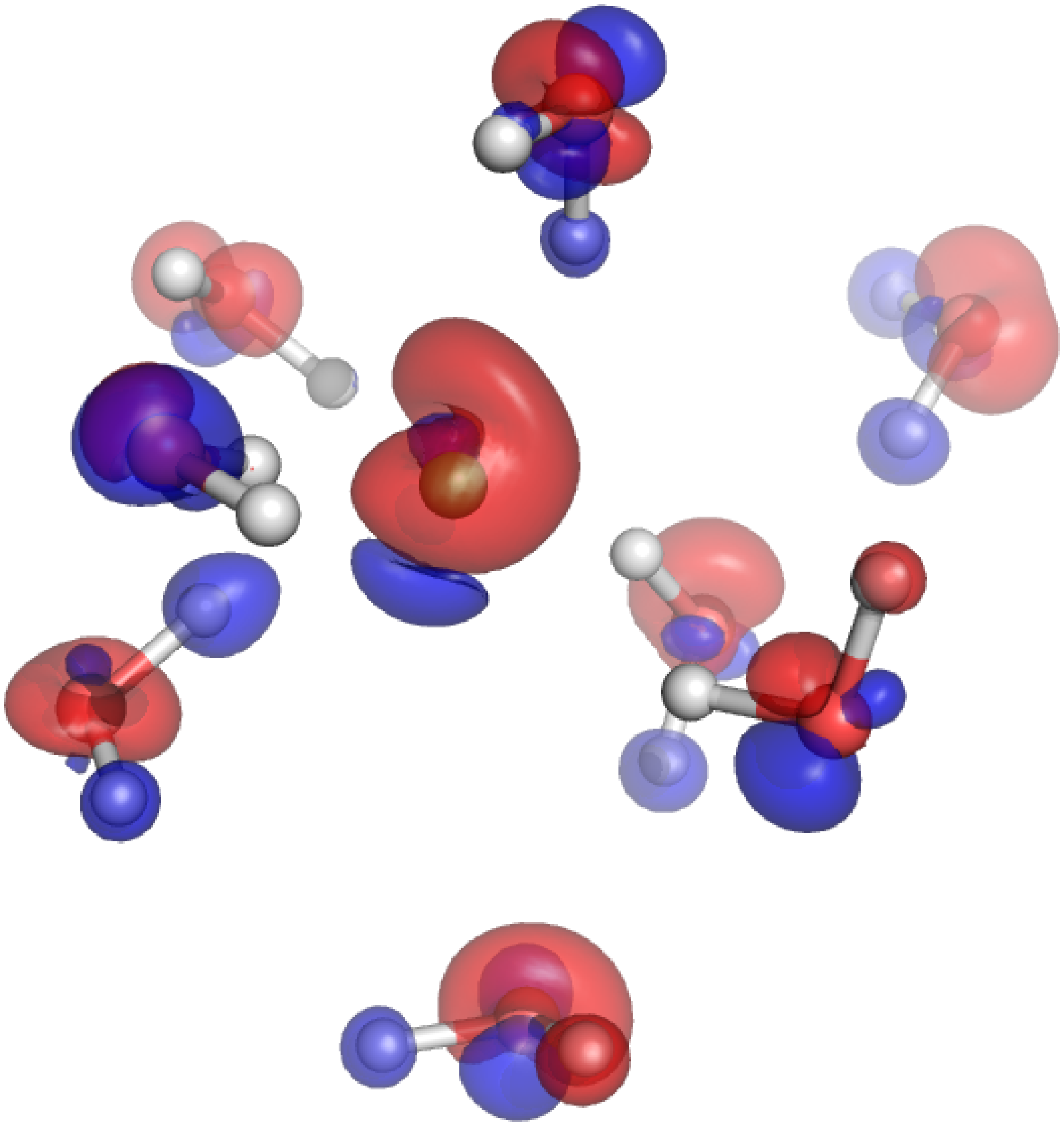

Figure S2. Imaginary part of the Fourier Transform of the TD induced density, $(\mathrm{Im}[\delta\tilde{\rho}(\boldsymbol{r},\omega)])$ corresponding to the excitation frequency $\omega = 668.586$ eV for the $[F(H_2O)_8]^-$ complex using rt-BOMME.

1.3 Spatial component of $S(\omega)$

Figure S3. Details on the three main energy ranges for the cross-section contribution along the x-direction (S_x) of the K-edge spectra of F^- and Cl^-.

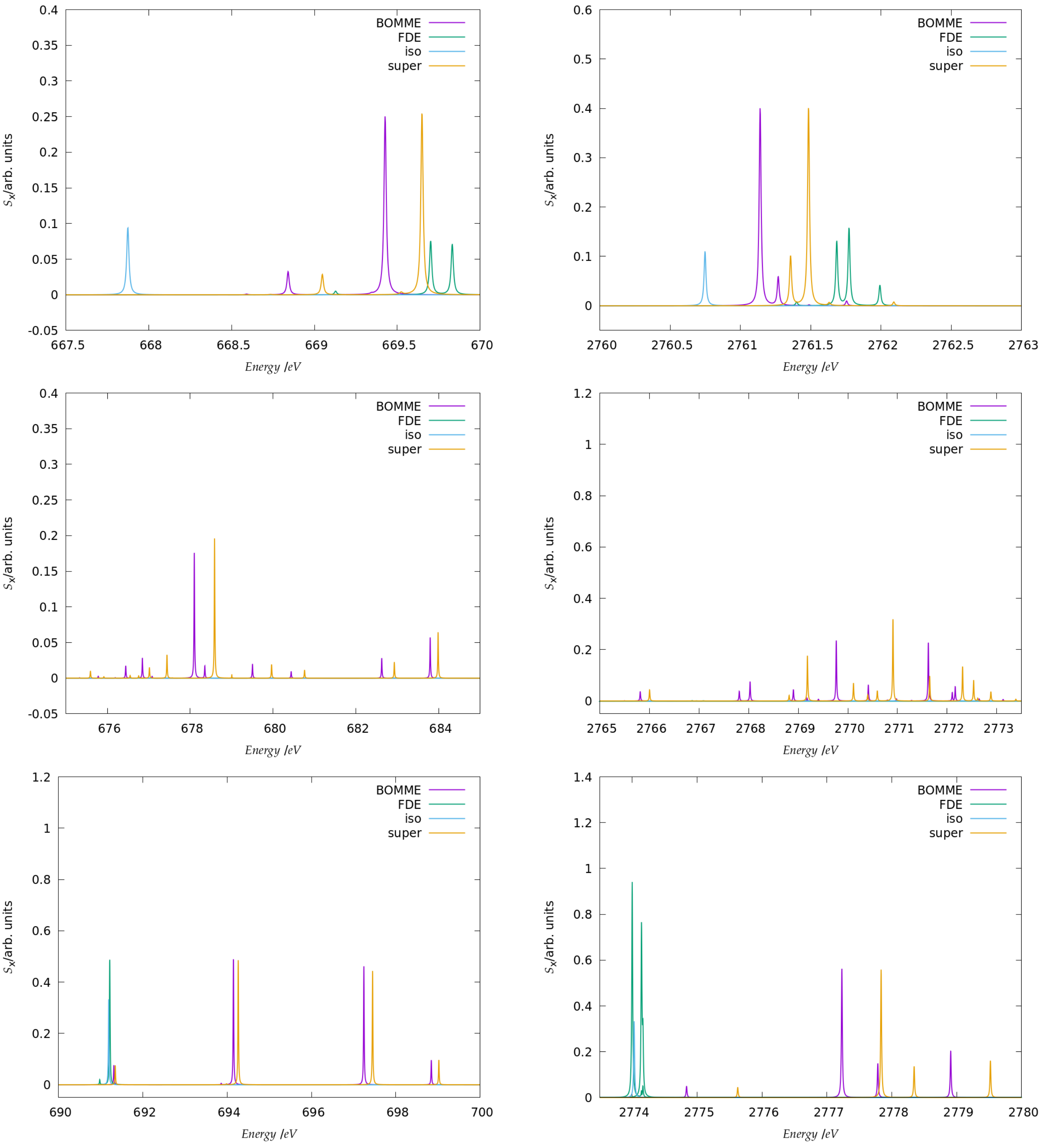

Figure S4. Details on the three main energy ranges for the cross-section contribution along the y-direction (S_y) of the K-edge spectra of F^- and Cl^-.

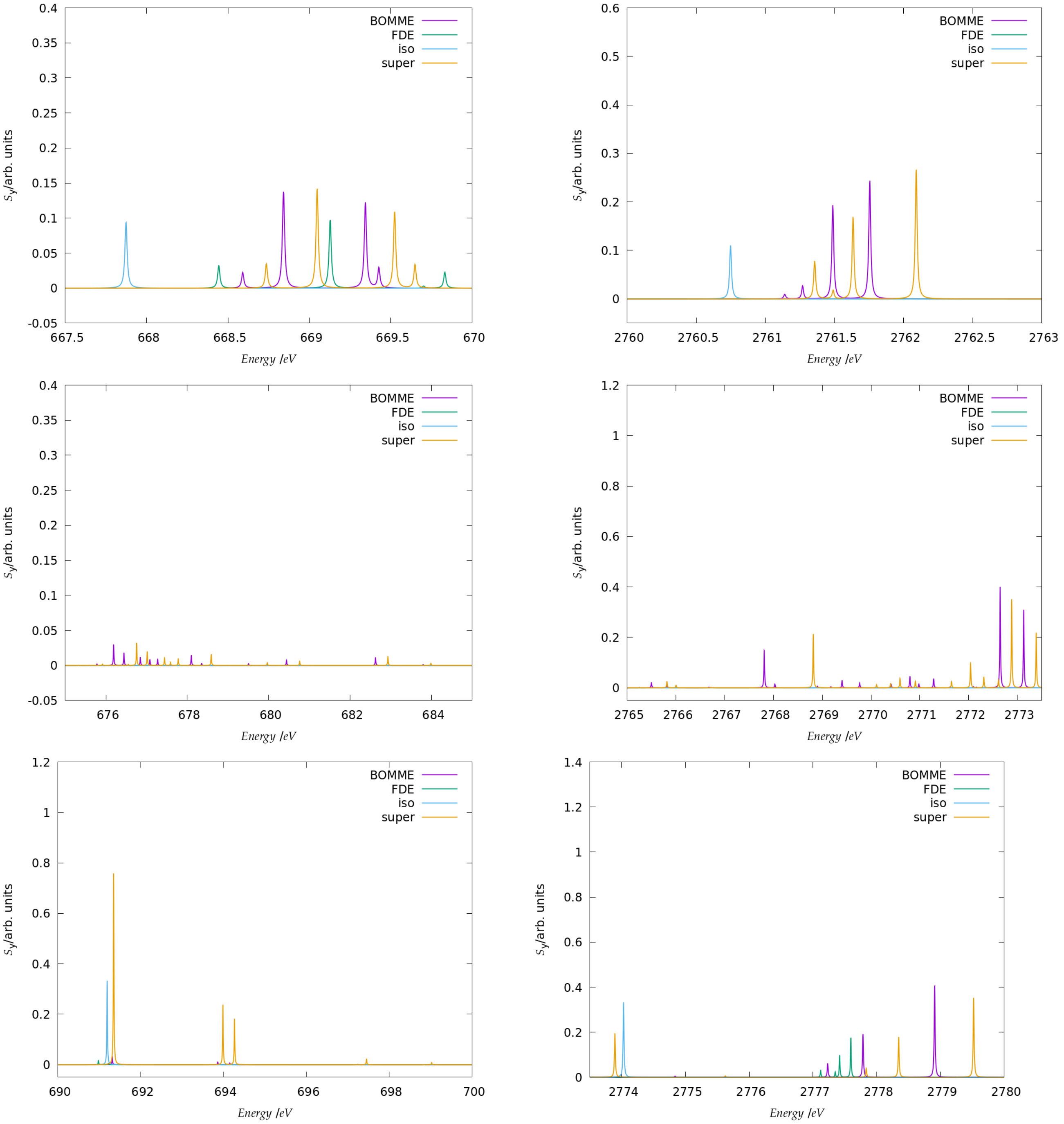

Figure S5. Details on the three main energy ranges for the cross-section contribution along the z-direction (S_z) of the K-edge spectra of F^- and Cl^-.

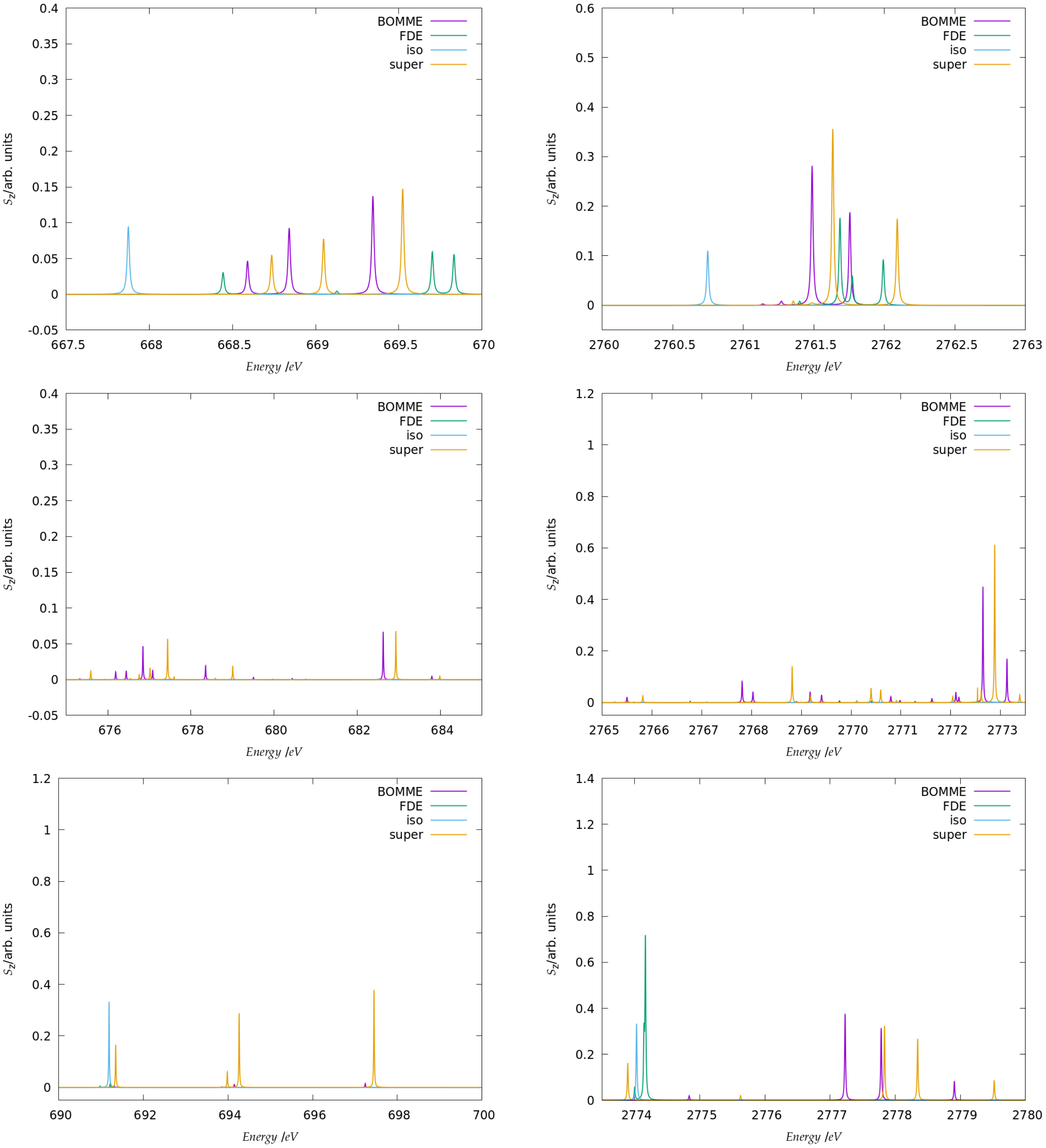

Figure S6. Details of the cross-section contributions along the x-,y-, and z-direction of the L_1-edge spectrum of Cl^-.

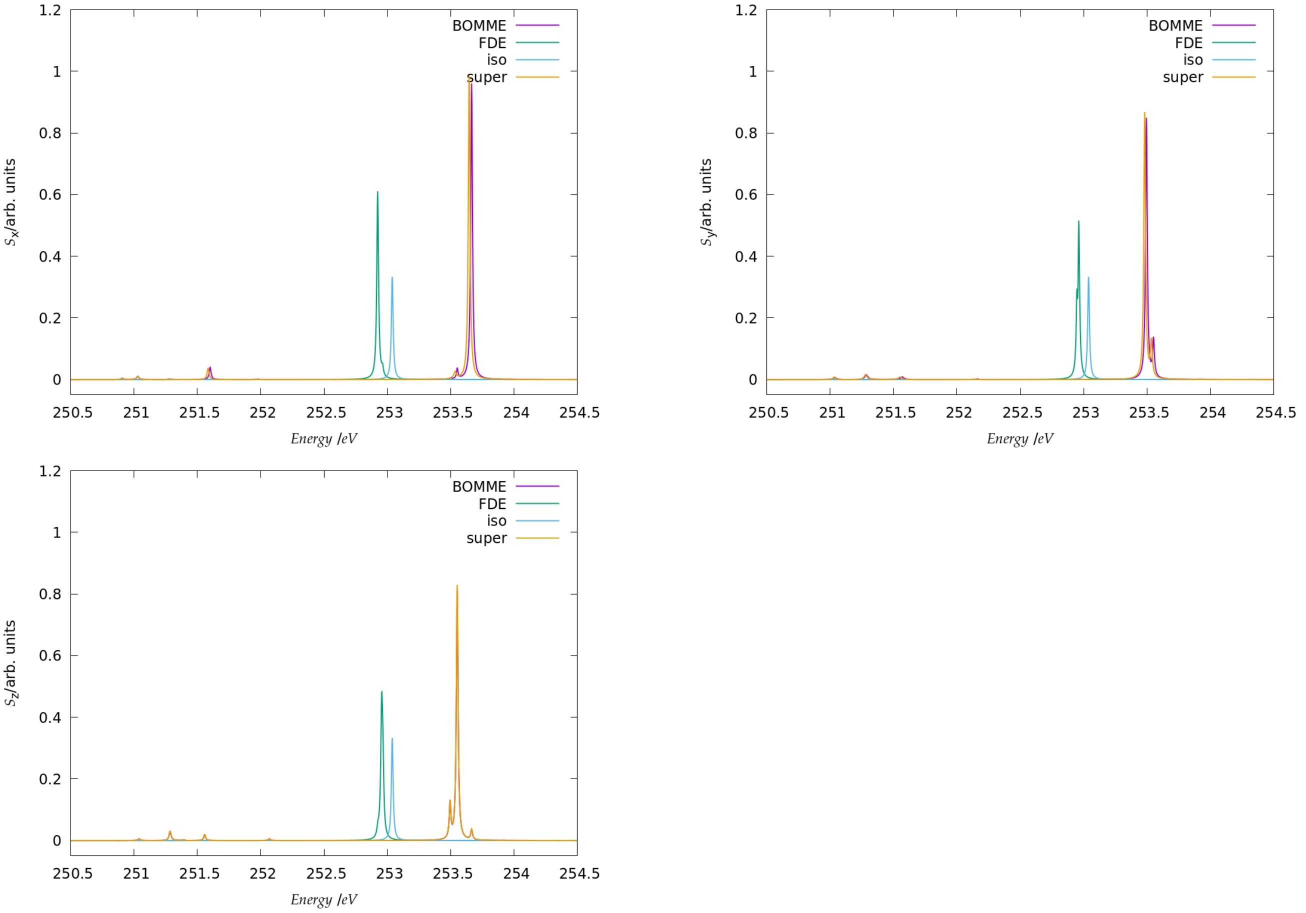

2 BASIS SET EFFECTS ON THE SPECTRA

In figure S7 we present a comparison of the z-component of the spectra (the same trends are observed for the other components) for lower energy section of the K edge spectra of fluoride and chloride, obtained with the aug-cc-pVTZ and aug-cc-pCVTZ basis sets.

We observe first that the improvement of the basis set in the core region, brought about by the additional tight functions in the aug-cc-pCVTZ sets, translates into shifts towards lower excitation energies for both fluoride (by slightly under 0.1 eV) and chloride (around 1 eV). In relative terms, these correspond however to variations of around 1% in the excitation energies.

The difference between the supermolecule and BOMME results, on the other hand, remains essentially the same for both basis sets, as can be seen from the $\Delta\omega$ values shown in each figure. This points to BOMME systematically reproducing the supermolecule calculations, irrespective of the basis set.

We note, however, that the improvement of the basis set doesn't introduces any change in the shape of the supermolecular spectra of fluoride, whereas for chloride two extra peaks appear. In both cases, no changes are observed for BOMME.

In order to investigate the origin of these peaks, we carried out rt-TDDFT calculations for the supermolecular system within the selected perturbation scheme proposed by Kadek et al. (2015), in which we only consider the transition density matrix elements between the 1s of chloride and the virtuals. In the corresponding spectra, shown in blue, we observe that only two peaks remain, indicating the others do not arise from K-edge transitions.

Given that the only difference between the BOMME and supermolecule calculations resides in the use of a different functional for the environment, we posit BOMME spectra are accidentally freed from such undesirable transitions, and that beyond the first-row, the selective perturbation scheme should become indispensable for obtaining reliable spectra when employing extended basis sets.

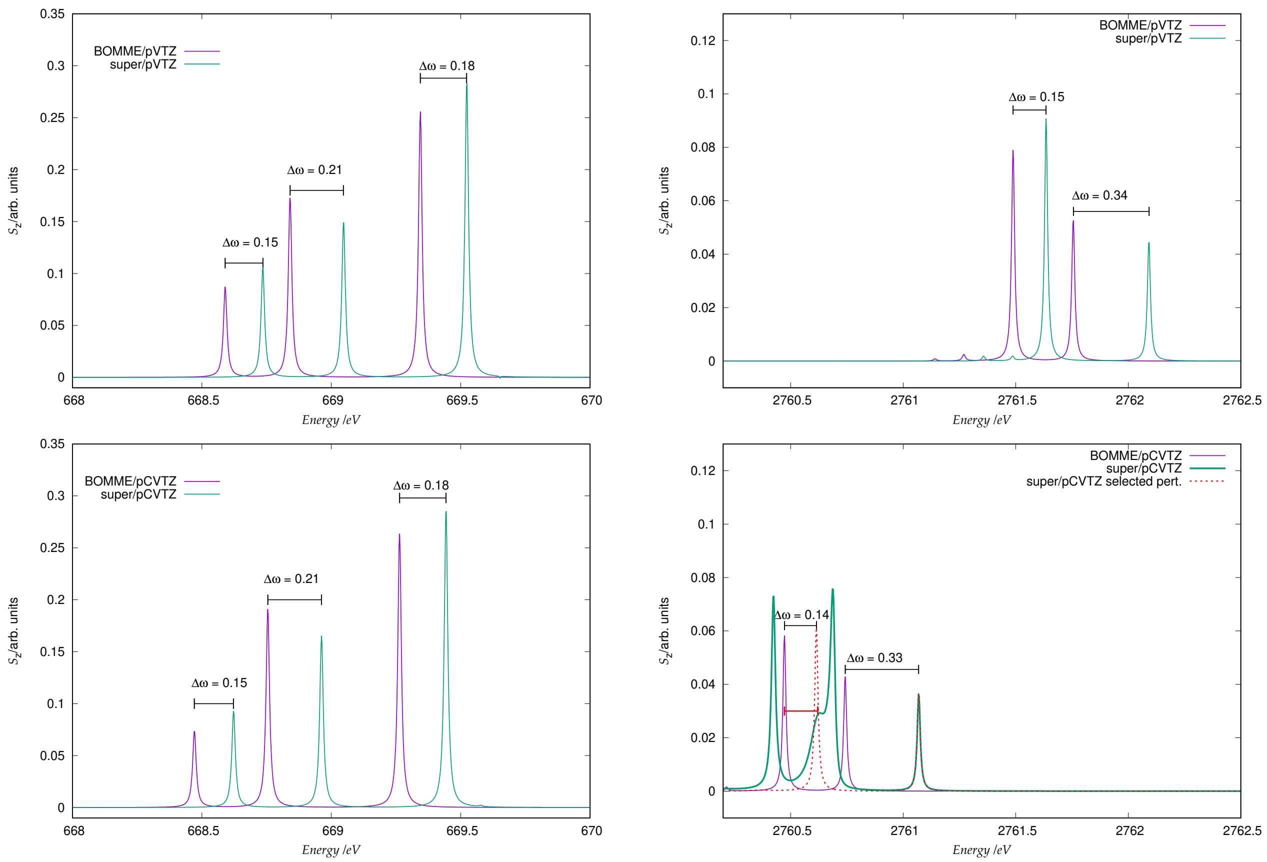

Figure S7. Comparison of the z-component of the spectra obtained with aug-cc-pVTZ (top) and aug-cc-pCVTZ (bottom) basis sets for BOMME and supermolecule (super) calculations for the fluoride (left) and chloride (right) K edges. For chloride, we also present results for calculations with the selected perturbation scheme of Kadek et al. (2015) (blue) for the aug-cc-pCVTZ set.

3 THE PERFORMANCE OF EMBEDDING APPROACHES

In figure S8, we present a comparison of the total time to solution (TTS) of the real-time calculations (thus excluding the SCF step). Since the Psi4NumPy implementation is OpenMP-parallelized, in this figure we also display how the TTS varies with increased number of cores used in the calculations.

We note first that BOMME consistently more efficient than the supermolecular calculations, with speedups of around 33%, which are due to the treatment of the environment with a lower-level theory. In addition to it, FDE calculations are much more efficient than both BOMME and supermolecule, since only the basis sets for the active subsystems are involved. Here, it should be noted that the FDE timings show a constant time due to the fact that, as discussed by De Santis et al. (2020), there is a significant overhead due to the transformation between the representation of the embedding potential over a grid to its matrix form at each time-step, that for this particular system dwarfs all other steps in the calculation.

Second, we see that there is an important speedup when going from 2 to 4 OpenMP threads, but that for a larger number of cores little performance improvement is seen, something that we attribute to the relatively small problem size in our case. In any case, given that that both BOMME and supermolecular calculations.

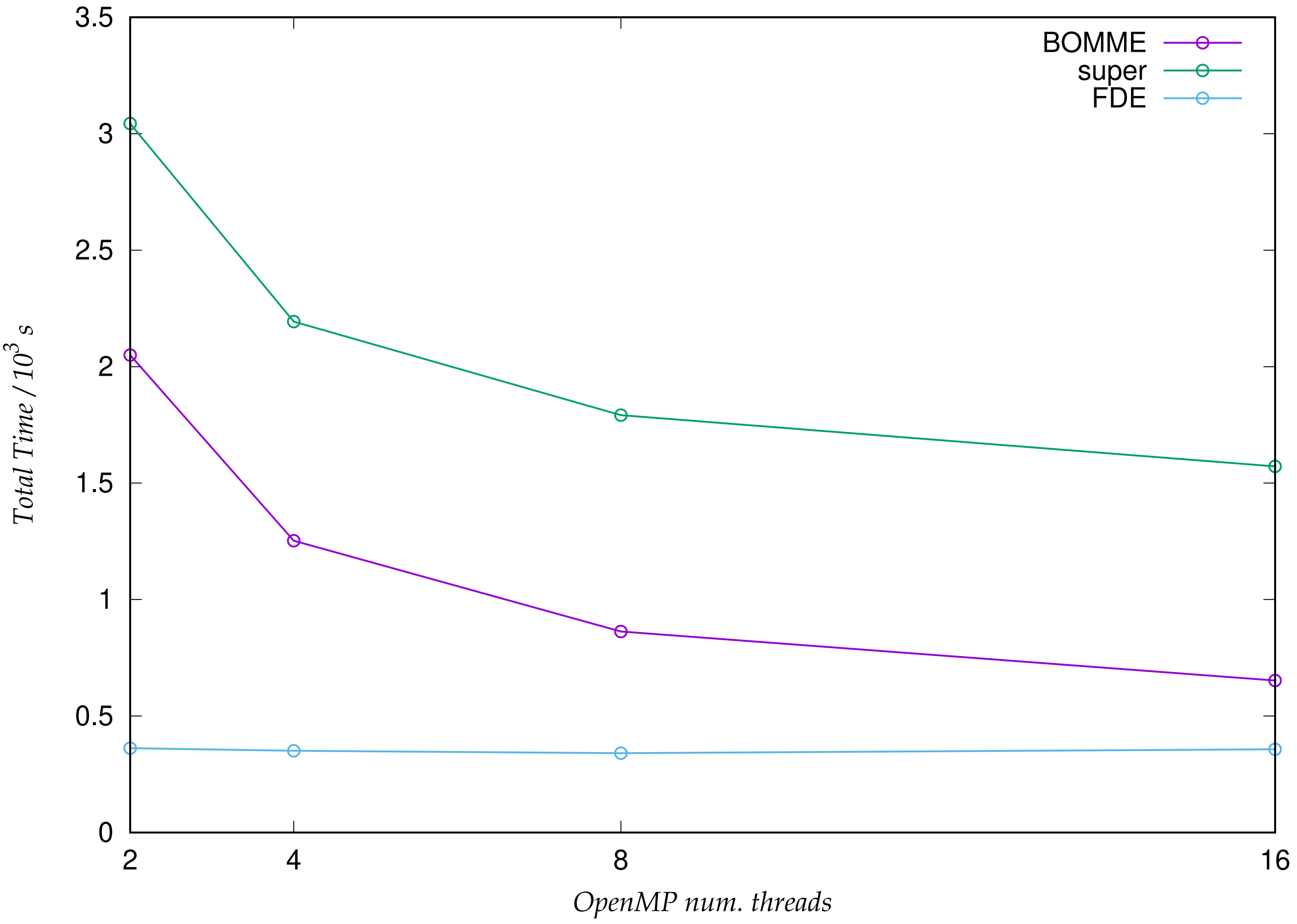

Figure S8. Comparison of times to solution (TTS, in seconds) of the real-time simulations with aug-cc-pVTZ basis sets for FDE, BOMME and supermolecule (super) calculations, as a function of OpenMP threads.

REFERENCES

Kadek M, Konecny L, Gao B, Repisky M, Ruud K. X-ray absorption resonances near l2,3-edges from real-time propagation of the dirac-kohn-sham density matrix. *Phys. Chem. Chem. Phys.* **17** (2015) 22566–22570. doi:10.1039/C5CP03712C.

De Santis M, Belpassi L, Jacob CR, Gomes ASP, Tarantelli F, Visscher L, et al. Environmental effects with frozen-density embedding in real-time time-dependent density functional theory using localized basis functions. *J. Chem. Theory Comput.* **16** (2020) 5695–5711. doi:10.1021/acs.jctc.0c00603.
